# Fabrication of CH_3_NH_3_PbI_3_/PVP Composite Fibers via Electrospinning and Deposition

**DOI:** 10.3390/ma8085256

**Published:** 2015-08-21

**Authors:** Li-Min Chao, Ting-Yu Tai, Yueh-Ying Chen, Pei-Ying Lin, Yaw-Shyan Fu

**Affiliations:** 1 Department of Greenergy, National University of Tainan, Tainan 70005, Taiwan; E-Mails: liminchao@gmail.com (L.-M.C.); mucy6331@gmail.com (T.-Y.T.); felicidad@livemail.tw (Y.-Y.C.); 2 Department of Photonics, National Cheng Kung University, Tainan 70005, Taiwan; E-Mail: sherry80128@gmail.com

**Keywords:** polyvinylpyrrolidone, perovskite, electrospinning

## Abstract

In our study, one-dimensional PbI_2_/polyvinylpyrrolidone (PVP) composition fibers have been prepared by using PbI_2_ and PVP as precursors dissolved in *N,N*-dimethylformamide via a electrospinning process. Dipping the fibers into CH_3_NH_3_I solution changed its color, indicating the formation of CH_3_NH_3_PbI_3_, to obtain CH_3_NH_3_PbI_3_/PVP composite fibers. The structure, morphology and composition of the all as-prepared fibers were characterized by using X-ray diffraction and scanning electron microscopy.

## 1. Introduction

Electrospinning is a novel and simple synthesis method for one-dimensional nanostructures. It stretches the solution using electrostatic forces and further spins it into a solid state. The fiber formed by electrospinning can achieve nano fineness and nanostructure surfaces. Thus, it can have different reactions with substances compared with macro materials [1]. One-dimensional nanofibers can promote charge transfer and reduce the recombination of the hole-electron pairs better than nanoparticles [2]. Basically, nearly all soluble or fusible polymers can be processed into fibers by electrospinning, provided that the molecular parameters and the process parameters are correctly adjusted [3]. Hereinto, polyvinylpyrrolidone (PVP) is a polymer containing lactam rings by which the polymer/metal-ion complexes can be formed. It is an important synthetic polymer with good complexation and adhesion properties, excellent physiological compatibility, low chemical toxicity, and reasonable solubility in water and most organic solvents, such as DMF, ethanol, and dichloromethane [4]. A lot of papers have reported the fabrication of electrospun composite fibers of PVP and metal compounds. Metal compounds are normally used in the form of sol-gel precursors, and the PVP fibers are used as templates to load the inorganic precursors [3]. Thereby, PVP was used for our study.

Lead iodide (PbI_2_) is a high anisotropic semiconductor. It crystallizes in the CdI_2_ type of layer structure, composed of a hexagonal close packing of iodine ions with the small lead ions intercalated between alternate layers of iodine [5]. It is an intrinsic wide band gap semiconductor, and so has potential applications such as in room temperature photocells, X-ray imaging, gamma-ray detectors, and photovoltaic [6]. A number of research works have been reported on the synthesis of the PbI_2_ with different nanocrystals and different morphologies, such as nanorods [7], nanotubes [8], nanowires [9], nanosheets [10], nanoplatelets [11], hollow spheres [12], nanospheres [13] by chemical and physical methods, including liquid crystal template [7], self-assembled growth [8], vapor-liquid-solid process (VLS) [9], solvothermal synthesis [10,11], argon transport method [11], hydrothermal method [13]. To the best of our knowledge, there have not been any reports on the synthesis of PbI_2_ fibers.

Perovskite is a common metallo-organic compound crystal, which is composed mainly of calcium titanium oxide (CaTiO_3_). It generally refers to a compound similar to the crystal structure of CaTiO_3_, with generic chemical formula ABX_3_. Organic-inorganic hybrid perovskite materials which meant including inorganic atoms and organic molecular clusters have been extensively investigated due to their excellent optical, electrical and mechanical properties [14]. Recently, methylammonium lead halide perovskites, CH_3_NH_3_PbX_3_ (X = Cl, Br, I), have demonstrated impressive progress as a new category of semiconductor light absorbers leading to solar cells with over 20.1% solar conversion efficiencies [15,16]. The CH_3_NH_3_PbI_3_ (MAPbI_3_) perovskite semiconductor has attracted attention because of the ease of solution processing and excellent absorption properties. It was often formed from the solution, such as γ-butyrolactone [17] and *N,N*-dimethylformamide (DMF) [18], containing equimolar mixture of methylammonium iodide (CH_3_NH_3_I, MAI) and PbI_2_ by coprecipitation method using spin coating technique. In addition, Chen *et al.* reported a one-pot solvothermal approach to synthesize the cuboid shaped MAPbI_3_ single crystals [19]. However, in our study, one-dimensional PbI_2_/PVP composite fibers have been prepared via an electrospinning process and then immersed in MAI solution to form perovskite MAPbI_3_/PVP composite fibers. The preparation method was similar to that which is used to prepare PAN/ZnS composite nanofibers [20].

## 2. Results and Discussion

### 2.1. PbI_2_/PVP Composite Fibers

**Figure 1:**
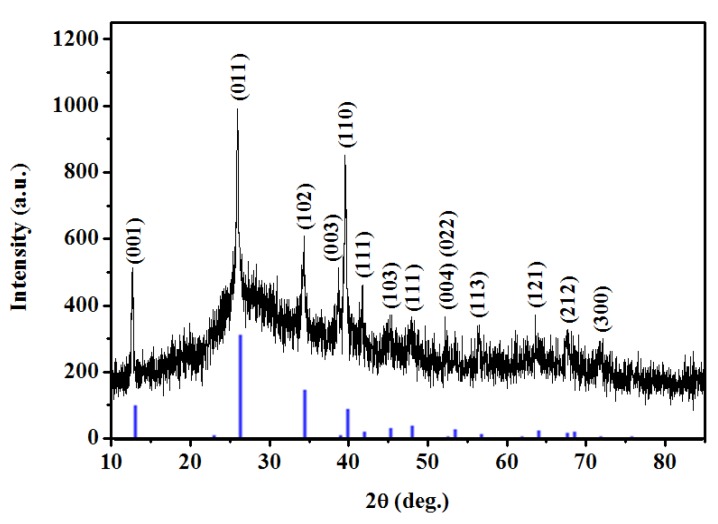
XRD diagram of PbI_2_/polyvinylpyrrolidone (PVP) composite fibers obtained from the electrospinning experiment using a precursor solution of 4.55 wt% PVP with 16 kV applied voltage, 10 cm spinning distance and flow rate of 1 mL/h. The Blue peaks are from JCPDs No. 80–1000 of PbI_2_.

An X-ray powder diffraction (XRD) analysis is conducted on PbI_2_/PVP composite fibers produced by electro spinning, with the results shown in Figure 1. The indicated peak complies with the multiple crystalline phases of PbI_2_ (JCPDs No. 80–1000). It was found that PbI_2_ powder forms a composite fiber with PVP after dissolution and electrospinning. Without a high-temperature and high-voltage process, PbI_2_ undergoes phase changes, so that its structure does not change.

The working parameters of electrospinning included solution parameters, process parameters and ambient parameters, which did not only influence the electrospinning process, but also affected the transformation of polymeric precursor solutions into fibers. The synthesis experiment of PbI_2_/PVP composite fibers explored the changing polymer concentration (C) among solution parameters, applied voltage (V) of process parameters, spinning distance (L), and flow rate (FR).

**Figure 2:**
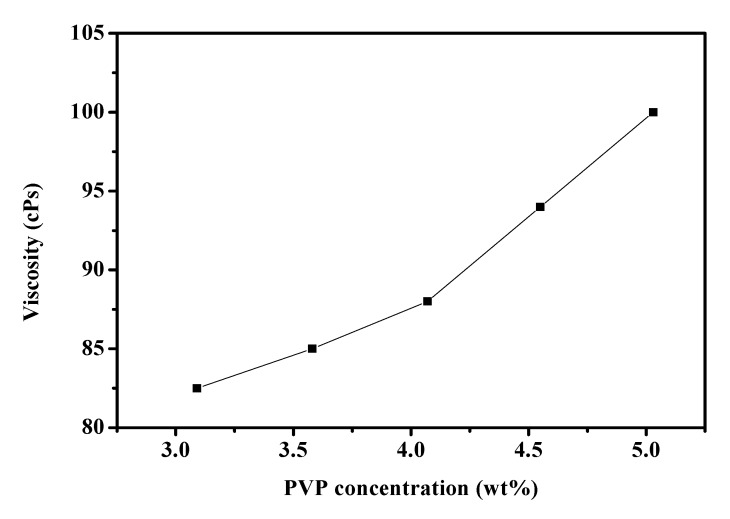
The changes in viscosity of PbI_2_/PVP precursor solutions with its concentration.

**Figure 3:**
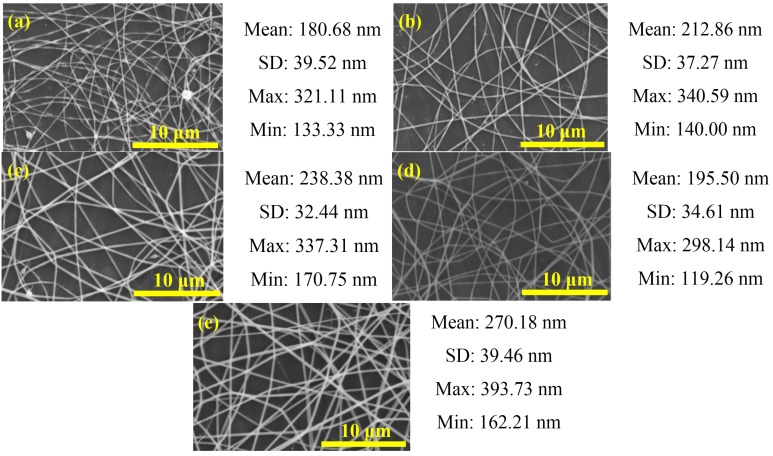
Scanning electron microscopy (SEM) images of PbI_2_/PVP composite fibers produced from precursor solutions with different PVP concentration: (**a**) 3.08; (**b**) 3.58; (**c**) 4.07; (**d**) 4.55 and (**e**) 5.03 wt% (V = 15 kV, L = 10 cm, FR = 1.0 mL/h). The figure also shows the mean, standard deviation (SD), maximum and minimum values of the fiber diameter.

**Figure 4:**
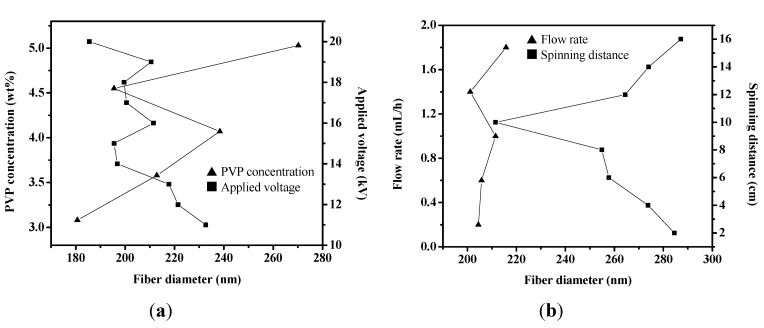
The average diameter tendency chart of PbI_2_/PVP composite fibers produced by changing the working parameters of electrospinning: (**a**) PVP concentration and applied voltage; (**b**) spinning distance and flow rate.

The polymer concentrations of precursor solution play an important role in the fiber formation during the electrospinning process [2]. The morphology and size of electrospun nanofibers depend on solution properties such as viscosity. Concentration is a primary factor determining the solution viscosity [4]. We used the precursor solutions with 3.08–5.03 wt% PVP, 15 kV applied voltage, 10 cm spinning distance and flow rate of 1.0 mL/h during the electrospinning process to synthesize fibers, and we observed the effect of polymer concentration on fibers. The viscosity of the PbI_2_/PVP precursor solutions was about 82.5 to 100 cPs (Figure 2). The diameter of the output fibers is about 110–400 nm and the apperance is smooth without beads (Figure 3). And, the average diameter and the viscosity of the precursor solutions under higher PVP concentration tends to increase (Figure 4a). This is in agreement with Chen *et al.* [21], who mentioned in the electrospinning experiment on PVP fiber production that, with the increase of polymer PVP concentration in the precursor solution, the solution viscosity would become higher, making the fiber surface smooth without beads. This would also be helpful to the output of the fibers given the corresponding increase in average diameters [22]. However, the average diameter of the fibers produced from the precursor solution with 4.55 wt% PVP was small and uniform. It was also the smallest among the mean fiber diameters, which surpassed our expectation. As for the fibers produced from 3.08 wt% PVP, the fiber films could be easily obtained from the collection board in large pieces and clots. Thus, in succeeding steps, the precursor solutions with 4.55 wt% PVP were used.

**Figure 5:**
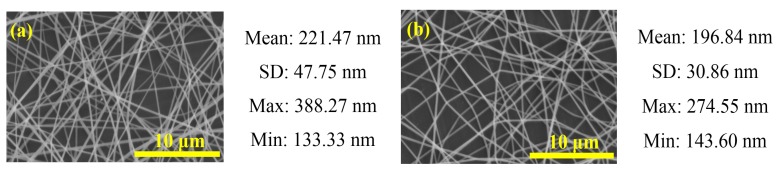

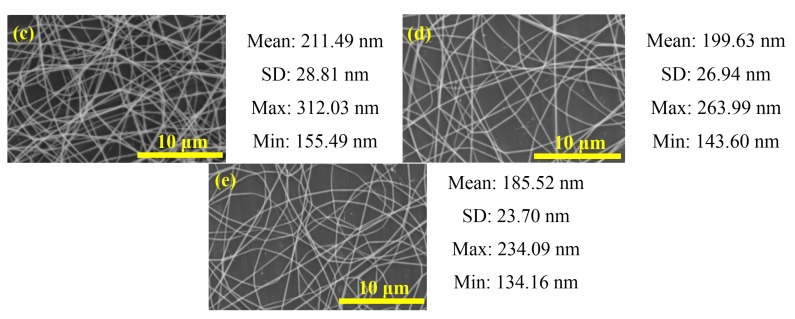
SEM images of PbI_2_/PVP composite fibers produced at different applied voltages: (**a**) 12; (**b**) 14; (**c**) 16; (**d**) 18 and (**e**) 20 kV (L = 10 cm, FR = 1.0 mL/h).

The applied voltage is a critical factor in electrospinning. When the applied voltage is higher than the threshold voltage, charged jets could be ejected from Taylor Cone [2]. In the electrospinning experiment published by Li and Xia [23], it was found that when applied voltage increases, the fiber diameter would be smaller. However, when voltage is higher than a certain value, the fiber diameter would be larger. Most studies supported these findings, but the applied voltage does not significantly influence the fiber diameter [22]. In order to observe whether applied voltage has significant influence on the formation of PbI_2_/PVP composite fibers, we designed different applied voltages with a fixed spinning distance of 10 cm and flow rate of 1.0 mL/h for fiber output during electrospinning. The diameter of the fiber with smooth apperance is about 130–390 nm (Figure 5). Based on Figure 4a, with an increase in applied voltage, the average fiber diameter tends to be smaller even though it does not show obvious and regular changes.

**Figure 6:**
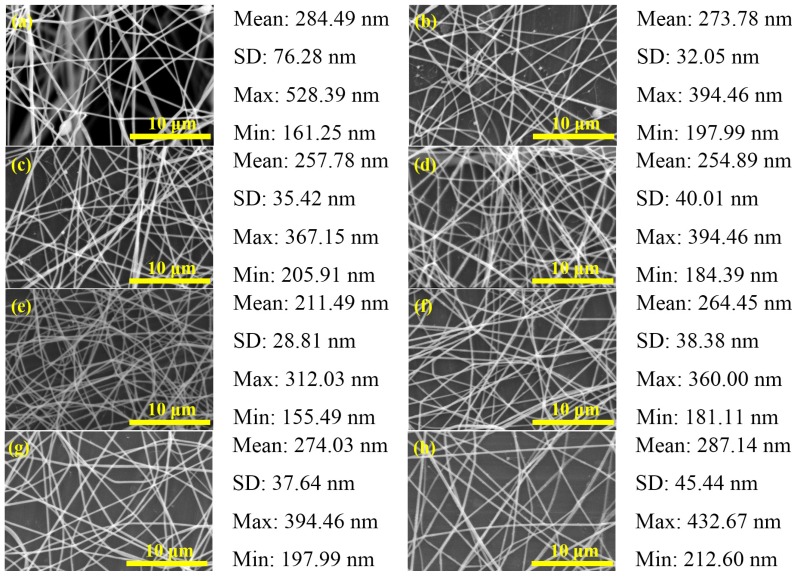
SEM images of PbI_2_/PVP composite fibers produced at different spinning distance: (**a**) 2; (**b**) 4; (**c**) 6; (**d**) 8; (**e**) 10; (**f**) 12; (**g**) 14 and (**h**) 16 cm (V = 16 kV, FR = 1.0 mL/h).

In the electrospinning experiment of Lee *et al.,* it was found that changing the spinning distance between the needle tip and the collection board could obtain fibers with smaller diameters [24]. When the spinning distance is too short, the fiber would not have enough time to solidify. However, if it is too long, it would generate fibers with beads [2]. In this experiment, we used different spinning distances, applied voltage of 16 kV and flow rate of 1.0 mL/h to produce fiber. In the trend chart shown in Figure 4b and the SME diagram in Figure 6, the fiber diameter is between 150 and 530 nm. When the spinning distance continues to grow longer, the beads become less and less until they finally disappear. However, the fiber diameter becomes smaller first and gradually gets bigger.

**Figure 7:**
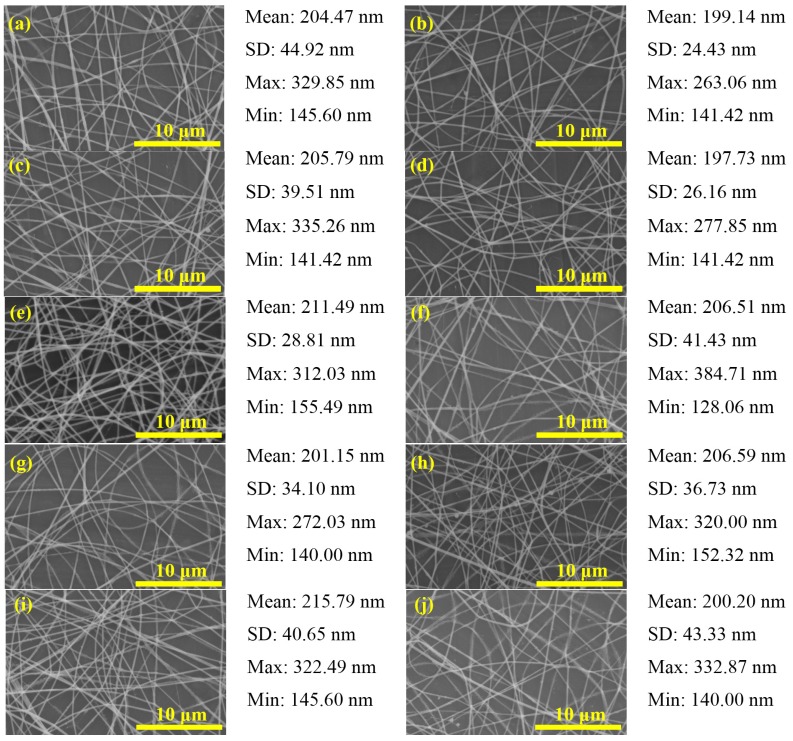
SEM images of PbI_2_/PVP composite fibers produced at different flow rate: (**a**) 0.2; (**b**) 0.4; (**c**) 0.6; (**d**) 0.8; (**e**) 1.0; (**f**) 1.2; (**g**) 1.4; (**h**) 1.6; (**i**) 1.8 and (**j**) 2.0 mL/h (V = 16 kV, L = 1.0 cm).

The flow rate of the precursor solution is a key process parameter in electrospinning, which leaves enough time for the precursor solution to polarize [2]. However, it does not have absolute influence on the size of the fiber diameter. Based on the findings of Li *et al.* [23] and Megelski *et al.* [25], when flow rate is increased, the fiber diameter becomes larger. However, according to the experimental results of Tan *et al.* [22], there are no significant changes. This experiment observed the influence of flow rate from fiber produced at different flow rates, 16 kV applied voltage and 10 cm spinning distance. The fiber diameter is about 140–390 nm with a smooth apperance, but the beads are gradually produced under high flow rate (Figure 7). Based on Figure 4b, the fiber diameter shows no significant change under higher flow rate.

To sum up the above experiments for each parameter, we can deduce that PbI_2_/PVP composite fibers produced under an applied voltage of 16 kV, spinning distance of 10 cm and flow rate of 1.0 mL/h, have small and uniform average fiber diameter (with small standard deviation), and smooth surface without beads. Thus, such fibers are used as base material to synthesize MAPbI_3_/PVP composite fiber.

### 2.2. MAPbI_3_/PVP Composite Fibers

**Figure 8:**
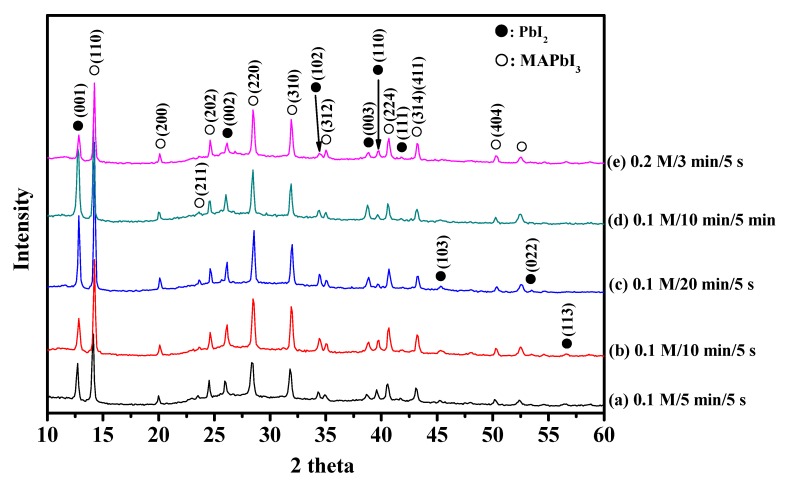
XRD images of MAPbI_3_/PVP composite fibers produced at different MAI/IPAconcertaion/immersing time/IPA rinsing time.

**Figure 9:**
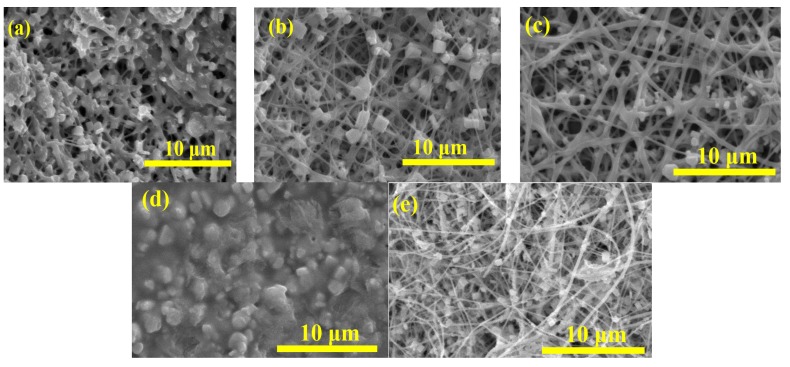
SEM images of MAPbI_3_/PVP composite fibers produced at different MAI/IPA different MAI/ isopropyl alcohol (IPA) concertaion/immersing time/IPA rinsing time: (**a**) 0.1 M/5 min/5 s; (**b**) 0.1 M/10 min/5 s; (**c**) 0.1 M/10 min/5 min; (**d**) 0.1 M/20 min/5 s and (**e**) 0.2 M/3 min/5 s.

PbI_2_/PVP composite fibers were immersed in 0.1 M or 0.2 M MAI solution for 3–20 min, and rinsed with IPA for 5 s or 5 min to form MAPbI_3_/PVP composite fibers. Afterwards, an XRD test was conducted to obtain the results shown in Figure 8. JCPDs No. 80–1000 was used for PbI_2_ while [26] was used for MAPbI_3_ as reference for comparison. It was observed that the fibers generated MAPbI_3_ which was still mixed with PbI_2_. This was probably because of the PbI_2_ scattered in the PVP polymer fiber which did not react with MAI. As for the fiber appearance, the SME diagram (Figure 9) shows square particles generated on the fibers. A longer immersion time would generate larger particles and large fiber diameters in molten state. However, it was found that fibers produced when immersed in 0.1 M MAI/IPA solution for 10 min and rinsed with IPA for 5 s, were clearly visible and uniform in size (Figure 9c), which had less square particles than fibers immersed for 5 min and 20 min.

Although this experiment outputs perovskite MAPbI_3_ fibers, it could hinder the function of electrons when applied in the production of perovskite battery components due to the existence of PbI_2_ and PVP. Thus, it is important to correct the formation of MAPbI_3_ fibers. In addition, Znao and Zhu investigated the impact of a NH_3_ gas environment on the structural and optical properties of MAPbI_3_ [15]. Therefore, we will try to show that the MAPbI_3_/PVP composite fibers can potentially be used as ammonia sensors with both a fast response time and a wide range of spectral responses in the future.

## 3. Experimental Section

### 3.1. Materials

PVP (average *M_w_* = 1,300,000, Acros Organics, Morris Plains, NJ, USA), PbI_2_ (100%, Showa Chemical, Tokyo, Japan), DMF (99.9%, Fisher Scientific, Fair Lawn, NJ, USA), methylamine (40 wt% in methanol, Panreac Sintesis, Barcelona, Spain), hydroiodic acid (57 wt% in water, Showa Chemical, Tokyo, Japan), isopropyl alcohol (IPA, 99.9+%, Burdick & Jackson, Ulsan, Korea), diethyl ether (99%, Echo Chemical, MiaoLi, Taiwan) were used as received, without further purification.

### 3.2. Synthesis of PbI_2_/PVP Composite Fibers

Exactly 2.71 mmol (1.248 g) of PbI_2_ was stirred to dissolve in 3 mL of DMF by magnetic force at 70 °C, and then 0.18, 0.21, 0.24, 0.27, 0.30 g PVP in 3 mL of DMF was tardily dropped into it at room temperature. The homogeneous PbI_2_/PVP composites precursor solutions with 3.08, 3.58, 4.07, 4.55, 5.03 wt% PVP were obtained.

**Figure 10:**
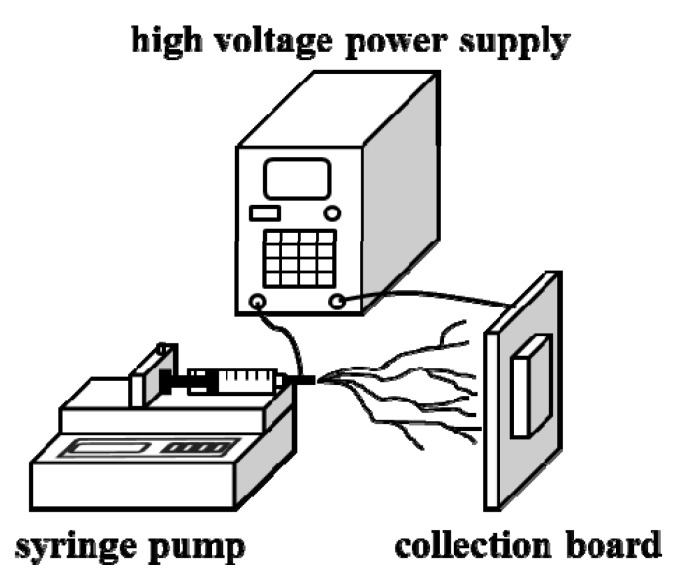
Electrispining device diagram.

Take 10 mL of the precursor solution into a 26-gauge syringe (with 0.82 mm diameter), which is fixed on a spring pump. The flow rate of the liquid is 0.2, 0.4, 0.6, 0.8, 1.0, 1.2, 1.4, 1.6, 1.8, 2.0 mL/h. A high-voltage power supply is connected to the needle tip from one end and to the collection board on the other end. The applied voltage is 11, 12, 13, 14, 15, 16, 17, 18, 19, 20 kV. The needle tip-to-collector distance is the spinning distance, which is 2, 4, 6, 8, 10, 12, 14, 16 cm. Since high voltage was applied, a drop of the precursor solution on the tip of the syringe would become polarized. In a high voltage electric field, the initial semi-circle drops form a Taylor cone. After the repulsive force between the electric charges becomes larger than the tensile force on the surface, the bottom of the Taylor cone would eject the charged jets. In the electric field, the jets usually pass by in a nearly straight line after bending into a complex path. When the jets are in the air, the volatile solution undergoes volatilization. Finally, the fiber films with a diameter at the nanometer level can be obtained on the collection board [2]. By drying the collected fibers at 80 °C, various kinds of PbI_2_/PVP composite fibers in different sizes can be obtained. The electrospinning setup used in this work is shown in Figure 10.

### 3.3. Synthesis of CH_3_NH_3_PbI_3_/PVP Composite Fibers

The MAI powder compounded based on the literature [17] is dissolved into the IPA blending solution with a concentration of 0.1, 0.2 M. The PbI_2_/PVP composite fibers are immersed into the blending solution for some time before taken out. The color of the fiber would be black, indicating that MAPbI_3_ is generated on the fiber. The fiber is then rinsed with IPA solution and dried at a temperature below 70 °C before finally obtaining MAPbI_3_/PVP composite fibers.

### 3.4. Characterization

The XRD patterns of the as-prepared samples were measured using a Rigaku MiniFlex II X-ray diffractometer (Tokyo, Japan) (*θ*/2*θ* geometry, Cu Kα radiation of λ = 0.15418 nm, graphite monochromator, scintillation counter, operating at 30 kV × 15 mA , step width 0.02°, scan rate of 4°/min, measured range of 10° to 85° in 2*θ*). The morphology of all composite fibers was observed using Hitachi S-3000N SEM (Tokyo, Japan) (operating at 15 kV).

## 4. Conclusions

In this work, PbI_2_/PVP composite fibers were successfully fabricated via electrospinning. We made the most suitable PbI_2_/PVP composite fibers with the fiber diameter about 140–390 nm under 4.55 wt% PVP concentration, 16 kV applied voltage, 10 cm spinning distance, and 1.0 mL/h flow rate of working parameters on electrospinning. After the most suitable PbI_2_/PVP composite fibers were dipped into 0.1 M MAI/IPA solution for 10 min, the better MAPbI_3_/PVP composite fibers could be obtained. Despite the MAPbI_3_/PVP composite fibers was not used as the sensitizer of solar cells, they will be able to as ammonia sensors.
